# Duration-dependent effects of social isolation on reintegration behavior in the swarming soldier crab, *Mictyris guinotae*

**DOI:** 10.1371/journal.pone.0350642

**Published:** 2026-07-01

**Authors:** Ruijia Yang, Kentaro Eto, Takumi Nakano, Yukio Pegio Gunji

**Affiliations:** Department of Intermedia Art and Science, Waseda University, Tokyo, Japan; IEAPM: Instituto de Estudos do Mar Almirante Paulo Moreira, BRAZIL

## Abstract

Social contact is fundamental to the behavioral and physiological homeostasis of group-living animals. When such contact is disrupted, even briefly, individuals may undergo behavioral shifts that persist beyond the isolation period and alter their interactions upon reintegration into the group. The soldier crab *Mictyris guinotae*, which forms large, dense swarms on intertidal flats, provides a tractable natural system for studying how transient isolation affects subsequent reintegration into collective behavior. Unlike eusocial insects with stable social roles, *M. guinotae* exhibits flexible, non-hierarchical interactions, allowing individual perturbations to be examined without confounding effects of dominance or task specialization. We conducted two experiments to examine how different durations of enforced isolation influence subsequent reintegration into collective movement. Isolation periods ranged from 5 to 180 minutes, and behavioral parameters such as local polarity, movement distance, and nearest-neighbor proximity were compared with controls. Individuals isolated for 5–30 minutes exhibited increased locomotor activity and reduced nearest-neighbor distance relative to controls, whereas 3-hour isolation induced a biphasic pattern characterized by an initial reduction in movement followed by elevated activity that remained weakly coordinated with the swarm. These results reveal a non-linear, threshold-dependent effect of isolation in a non-eusocial, anonymous collective. The study establishes *Mictyris guinotae* as a tractable invertebrate system for examining how transient social disruption alters measurable individual-level behavior and propagates through local interactions to shape emergent group-level dynamics.

## Introduction

Sociality confers substantial fitness advantages, including enhanced foraging efficiency, predator avoidance, and information transfer [1,2], but it also entails costs such as increased competition and resource sharing among group members [1,2].

However, these benefits depend on the continuous maintenance of group cohesion and coordination [3,4]. When social contact is disrupted, even briefly, individuals can exhibit measurable behavioral changes during the isolation period, such as altered locomotor activity or spatial positioning. Upon subsequent reintegration into the group, these isolation-induced changes may influence how individuals interact locally and re-enter coordinated collective movement [5,6].

Extensive work on mammals and humans has shown that isolation or perceived loneliness can activate stress and inflammatory pathways, impair cardiovascular and immune function, and increase risk for depression and dementia [7,8]. Neurobiological studies link these outcomes to altered corticostriatal and limbic activity and dysregulation of dopaminergic and serotonergic signaling [5,6]. Rodent and primate models confirm that even transient isolation elevates corticosterone, disrupts motivation, and changes prefrontal–amygdalar connectivity [9]. In eusocial insects such as ants, isolation has comparably strong consequences: individuals deprived of nest-mates exhibit reduced grooming and foraging activity, immune suppression, and shortened lifespan associated with oxidative stress [10,11]. These findings indicate that social contact functions as a key regulator of physiological and behavioral stability rather than merely a contextual factor.

In decapod crustaceans, social context has also been shown to modulate physiological state and behavior. For example, grouping behavior in fiddler crabs reduces water loss under intertidal conditions [12]. In crayfish, social experience influences neuromodulatory control of escape responses [13], and early-life social deprivation alters future agonistic behavior and growth trajectories [14,15]. These studies highlight that, even in non-eusocial arthropods, transient or developmental social isolation can have lasting behavioral and physiological consequences.

While isolation effects in eusocial insects with stable caste systems are well documented, studies in less hierarchical or weakly social species, such as Drosophila, have also reported isolation-induced behavioral changes. However, comparatively little is known about how transient isolation influences reintegration dynamics in organisms forming large, anonymous swarms, where collective coordination emerges from local interaction rules rather than fixed social hierarchies [3,16–18].

A key question is how isolation-induced changes in an individual’s behavior influence local interaction parameters—such as alignment and attraction—upon reintegration into the group, and how these local effects may in turn perturb group-level collective order [19–20]. However, whether these disruptions scale linearly with isolation time or instead follow threshold-like or biphasic functions remains largely unexplored.

This coexistence of individual autonomy and collective coherence has been explored using a range of theoretical frameworks that link local interaction rules with emergent group-level dynamics, including classical models of collective motion such as self-propelled particle systems [21] and interaction-based models of coordinated movement [2,3,16], as well as more recent approaches such as mutual anticipation models [22,23] and stochastic Bayesian–inverse-Bayesian (BIB) frameworks that describe how individuals adaptively revise internal hypotheses based on local interactions [24,25].

These frameworks predict that even small perturbations in an individual’s experiential history can propagate through local interactions and give rise to detectable changes at the collective level. This prediction provides a clear basis for empirical testing using controlled isolation experiments. Building on this theoretical framework, we experimentally examined how varying durations of enforced isolation affect the reintegration behavior of *M. guinotae.*

As a model system, the soldier crab *Mictyris guinotae* inhabiting Okinawan tidal flats performs striking “marching” movements in groups of tens to thousands of individuals [26,27]. Despite the large group size, collective coordination in this species emerges from local, neighbor-based interactions, such that perturbations introduced by a single individual can propagate through the swarm and influence group-level dynamics.

Unlike most schooling models that assume local velocity averaging [21,28–30], *M. guinotae* maintains cohesive yet flexible swarms where individuals move semi-independently [27]. This tendency has also been observed in starlings [31,32] and locusts, and it is now believed that velocity matching may not play a central role in the mechanism of swarm formation [33].

Previous studies in ants and other insects have shown that both isolation and post-isolation reintegration can significantly alter locomotor activity, interaction rates, and collective coordination [34–37].

We hypothesized that isolation induces time-dependent modulation of locomotor and social interaction parameters, producing (1) heightened approach and movement activity after short isolation and (2) impaired coordination and withdrawal after prolonged isolation. By quantifying local polarity, movement distance, and nearest-neighbor proximity, we identify behavioral thresholds to compare the relative effects of short and long isolation intervals. These findings link individual behavioral modulation with emergent collective organization, highlighting the resilience—and fragility—of social homeostasis across biological scales.

## Materials and methods

### Model animals and study site

**Fig 1:**
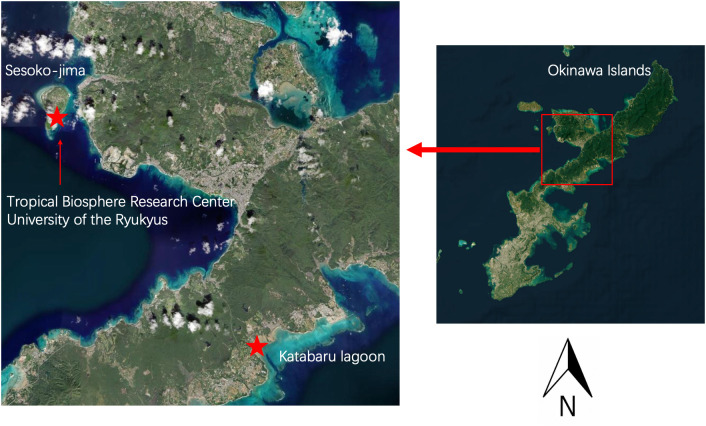
Location of the laboratory where the experiments were conducted and the field site (Katabaru lagoon) where crabs were collected. Map prepared by the authors using publicly available NASA Earthdata GIS resources and provided for illustrative purposes only.

*Mictyris guinotae* is a species of crustacean inhabiting tidal flats in subtropical regions of Japan, where they form large swarms of hundreds to thousands of individuals. Experiments were conducted at the Tropical Biosphere Research Center (TBRC), University of the Ryukyus, Sesoko-jima Island, Okinawa, Japan, from May 11 to May 16, 2022. Crabs were collected from the intertidal zone of Katabaru Lagoon (26°30'N, 127°59'E), Ginoza Village, Okinawa, during low tide. Collection was performed manually by multiple researchers over approximately 30 minutes. We selected active individuals with a carapace width of approximately 1.0–2.0 cm, specifically excluding any crabs with visible limb autotomy or signs of molting. Sex was not determined, as both male and female individuals participate indiscriminately in swarming behavior in this species. Following collection, all individuals were pooled in a common container and transported to the laboratory within 1 hour in shaded containers containing moist sand and ambient seawater to reduce desiccation and handling stress. Upon arrival at the laboratory, crabs were randomly assigned to experimental and control conditions. This randomization ensured that spatial proximity at the collection site, and any associated familiarity among near-neighbor conspecifics, did not systematically bias group composition across treatments (Fig 1).

### Ethical note

All experimental procedures complied with institutional and national guidelines for the ethical use of invertebrate animals in research. *Mictyris guinotae* is not a protected or regulated species in Japan, and no specific permits were required for collection or laboratory experimentation.

### Experimental setup and husbandry

Prior to experimentation, crabs were temporarily housed in plastic containers (30 cm in diameter) containing natural seawater and sand/silt substrate collected from the field site to allow burrowing behavior. Individuals were maintained at a stable room temperature of 27–31°C, consistent with ambient outdoor temperature conditions at the field site during the experimental period, under natural light conditions corresponding to the local day–night cycle. Seawater salinity was not artificially adjusted and reflected field conditions. Crabs were held at a moderate density (approximately 100 individuals within a circular container of 30 cm diameter, corresponding to approximately 1400 individuals per m²) and allowed to acclimate for approximately 2–6 hours following collection. The container was filled with approximately 10 cm of sand–mud substrate and ~3 cm of seawater.

**Fig 2:**
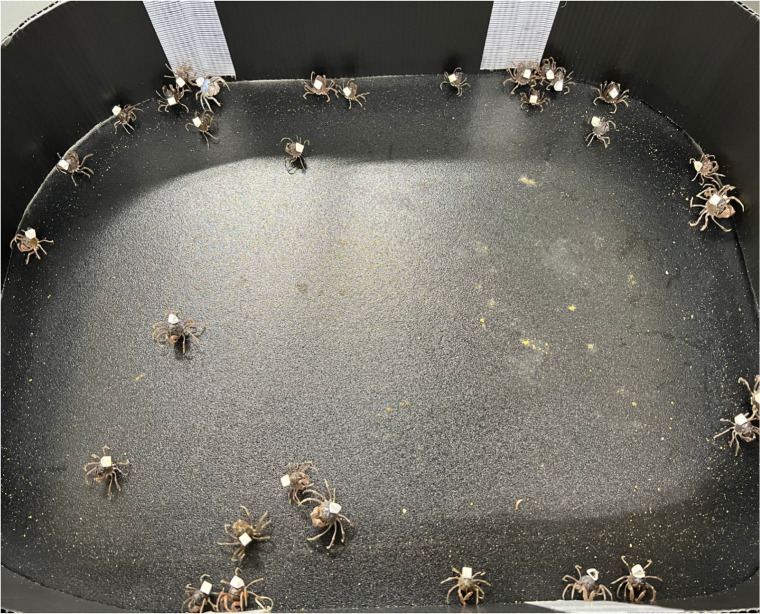
Experimental arena in which soldier crabs were placed and allowed to exhibit swarming behavior.

All experiments were conducted on the same day as collection and timed to coincide with the natural low-tide window (within ±2 hours of predicted low tide peak), during which the species is naturally active. During the holding and experimental periods, crabs were visually monitored; any damaged, molting, or inactive individuals were excluded prior to trials. No mortality was observed during the experimental period. The experimental arena was a 1.2 m × 1.2 m rectangular enclosure with a sand-covered floor. The walls were 50 cm high to prevent escape, and the arena was illuminated uniformly. Crabs were randomly assigned to either isolation or control groups. Social isolation was operationally defined as the complete visual deprivation of conspecifics. For isolation treatments, individuals were placed in white, opaque cylindrical containers (6 cm in diameter), ensuring no other crabs were visible. Control-group crabs were kept in a communal holding tank containing the same number of individuals as in the corresponding experimental condition (12 crabs in Experiment 1 and 20 crabs in Experiment 2) (Fig 2).

### Behavioral recording and tracking

Behavior was recorded using a high-definition video camera (Panasonic HDC-TM700) at 1920 × 1080 pixels and 60 fps. To facilitate tracking, small, white paper markers were attached to the dorsal carapace of the crabs. The position of each individual was then tracked using image processing software (Move-tr/2D ver. 8.31).

### Experimental procedure

#### Experiment 1: Disturbance in a swarm.

**Fig 3:**
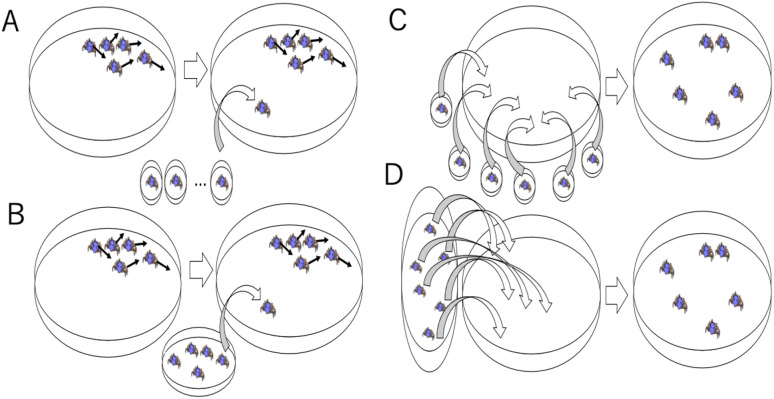
Schematic diagrams of the experimental procedures for Experiment 1 and Experiment 2. **(A)** Schematic overview of Experiment 1, illustrating the sequential introduction of isolated individuals. **(B)** Control experiment for Experiment 1 with no prior isolation. **(C)** Main experiment for Experiment 2, using the same procedure but with a different isolation duration condition. **(D)** Control experiment for Experiment 2. Although only six individuals are illustrated in the schematic, twenty individuals were used in the actual trials.

This experiment was used to determine whether a single formerly isolated individual could disturb an established swarm. In each trial, one crab was isolated in a 6 cm opaque container for a single, predetermined duration (5, 10, 15, 20, 25, 30, 35, 40, 45, 50, 55, or 60 minutes) and then reintroduced into the swarm. Each isolated individual experienced only one isolation duration and was used in a single trial. After the isolation period, this individual was reintroduced into a stable swarm, and the behavior of the group was recorded for 30 minutes (Fig 3A). Control trials involved introducing a non-isolated individual from the holding tank under identical conditions (Fig 3B).

#### Experiment 2: Formation of a of a swarm.

This experiment evaluated if a group of isolated individuals could form a swarm. Groups of 20 crabs were simultaneously isolated in individual 6 cm opaque containers for 5, 10, 30 minutes, or 3 hours. These durations span ecologically relevant timescales: shorter intervals (5–30 min) represent transient disruptions like swarm fragmentation, while the 3-hour interval represents prolonged separation. After the isolation period, all 20 individuals were released into the arena together, and behavior was recorded for 30 minutes (Fig 3C). Control groups consisted of 20 non-isolated crabs drawn from the same pooled housing conditions (Fig 3D).

### Data collection

Behavioral data were collected using high-definition video recordings at 60 frames per second.

Behavioral parameters were computed based on individual trajectories extracted at each frame.

Distance to the nearest neighbor was calculated at each time step (frame) as the Euclidean distance between an individual and its closest conspecific.

Local polarity was computed at each time step using velocity vectors estimated over a time interval τ, defined as the displacement between consecutive frames (τ = 1/60 s).

Movement distance over time was calculated as the cumulative distance traveled by each individual over a sliding window of 10 frames (approximately 0.17 s at 60 fps).

Behavioral differences between isolated and control groups were quantified using three metrics: distance to the nearest neighbor, local polarity, and movement distance over time. For each isolation duration, comparisons between the isolated (main) group and the corresponding control group were conducted using two-tailed unpaired t-tests.

Prior to statistical testing, data distributions were visually inspected and assessed for approximate normality. Independence of data points was ensured by treating each individual crab as a single observational unit, with no repeated measurements of the same individual across conditions.

Because comparisons were conducted independently for each behavioral metric and each isolation duration, with specific time segments defined a priori based on the experimental design, we treated each set of comparisons as a family and did not apply correction for multiple testing across families. All test statistics, degrees of freedom, and exact p-values are reported to allow readers to apply corrections if desired.

For all analyses, test statistics (t), degrees of freedom (df), and exact p-values are reported in the main text and figures. Statistical significance was defined as p < 0.05.

Formal mathematical definitions of the behavioral metrics are provided in S1 Methods.

## Results

### Experiment 1: Reintegration of a single isolated crab

#### Activity levels post-isolation.

**Fig 4:**
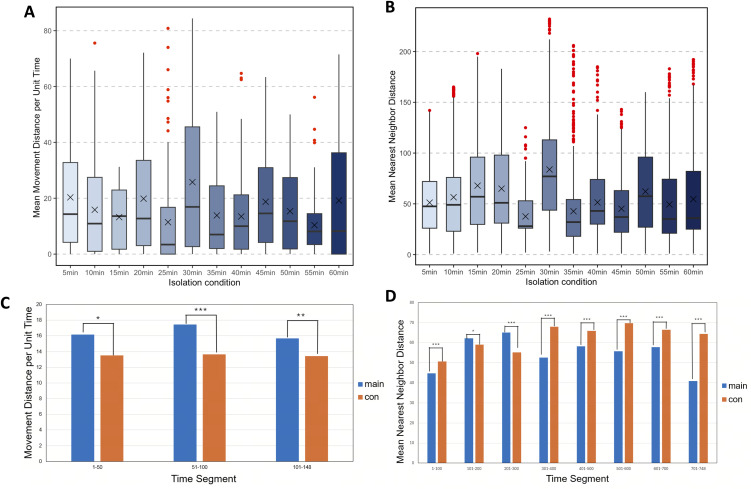
Moving distance and nearest-neighbor distance under different isolation durations. **(A)** Moving distance per unit time as a function of isolation duration, ranging from 5 to 60 minutes. **(B)** Distance to the nearest neighbor as a function of isolation duration. **(C)** Histogram comparing the average moving distance between isolated and control individuals. **(D)** Histogram comparing the average nearest-neighbor distance between isolated and control individuals.

Upon reintegration into an established swarm, formerly isolated individuals exhibited significantly higher movement activity compared to control individuals, whose activity levels remained relatively stable over the same period (Fig 4A).

#### Movement distance.

When reintroduced into a swarm, a single crab previously isolated for 5–60 minutes showed significantly greater activity than a non-isolated control crab (Fig 4A). This heightened movement was consistently significant across all analyzed time segments, such as the initial 1–50 segment (t(49) = 2.29, p = 0.026, Cohen’s d = 0.47), corresponding to an approximate 19.6% increase compared to the control group (Fig 4C) and the 51–100 segment (t(49) = 3.83, p < 0.001, Cohen’s d = 0.86), corresponding to an approximate 27.8% increase (Fig 4C). Even in the final 101–148 interval, the isolated individual remained significantly more active than controls (t(47) = 2.82, p = 0.007, Cohen’s d = 0.50), corresponding to an approximate 16.7% increase (Fig 4C) (see S1 Table for details).

#### Distance to nearest neighbor.

Reintegrated individuals demonstrated a strong tendency to seek social contact, maintaining significantly smaller distances to their nearest neighbors compared to control individuals (Fig 4B). This compensatory social seeking was highly significant during the first 100 time steps (t(99) = −5.80, p < 0.001, Cohen’s d = −0.27), corresponding to an approximate 11.6% reduction relative to controls (Fig 4D) and became even more pronounced during segments such as 301–400 (t(99) = −16.59, p < 0.001, Cohen’s d = −1.72), corresponding to an approximate 22.7% reduction (Fig 4D) and the final 701–748 interval (t(47) = −13.56, p < 0.001, Cohen’s d = −3.55), corresponding to an approximate 36.5% reduction (Fig 4D) (see S1 Table for details).

#### Local polarity.

**Fig 5:**
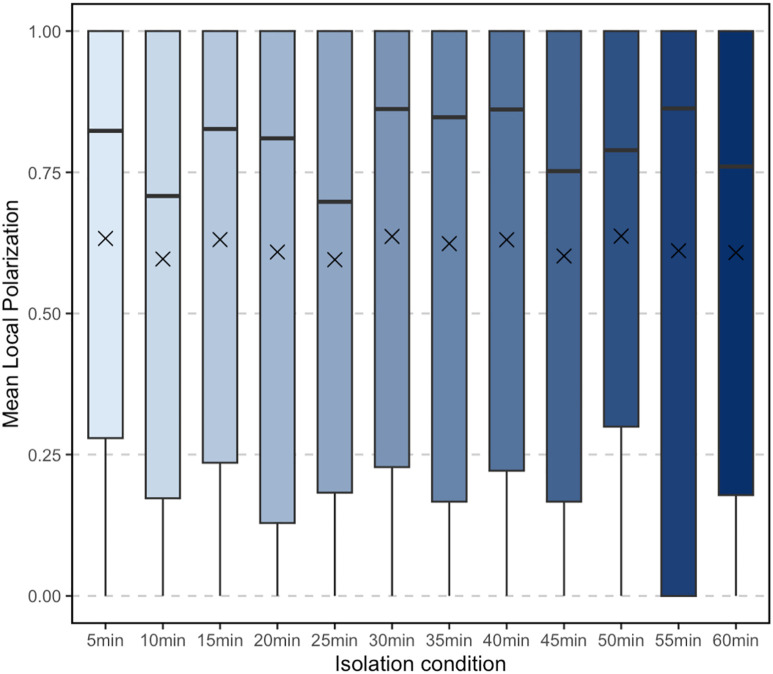
Local polarization of isolated individuals in Experiment 1 as a function of isolation duration (5–60 minutes).

**Fig 6:**
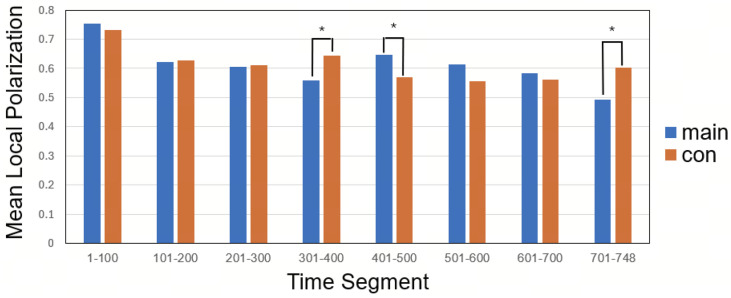
Comparison of the average local polarization between isolated individuals (Experiment 1) and non-isolated individuals (control).

The coordination of movement, measured by local polarity, revealed complex adjustments during reintegration (Fig 5). While initial alignment did not differ significantly from controls (1–100: t(99) = 0.54, p = 0.589, Cohen’s d = 0.08), corresponding to an approximate 2.9% increase (Fig 6), significant differences emerged in later segments, such as the 301–400 interval (t(99) = −2.29, p = 0.024, Cohen’s d = −0.33), corresponding to an approximate 13.3% reduction (Fig 6) and the 401–500 interval (t(99) = 2.52, p = 0.013, Cohen’s d = 0.34), corresponding to an approximate 13.7% increase (Fig 6), indicating a dynamic realignment phase during social re-entry.

### Experiment 2: Swarm formation by groups of isolated crabs

#### Activity levels post-isolation.

**Fig 7:**
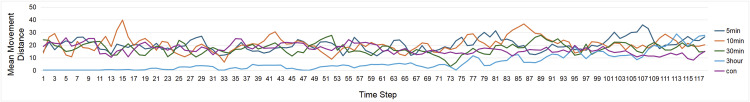
Time evolution of movement distance under different isolation durations and in the control condition. Curves are color-coded by isolation duration (5, 10, 30 minutes, 3 hours) and by the control condition.

During the 30-minute observation of group reformation, crabs generally exhibited a temporal increase in movement distance (Fig 7). However, isolation duration exerted a non-linear effect on subsequent activity levels.

#### Movement distance.

The duration of isolation had a significant and non-linear effect on the activity levels during swarm formation. A time-course analysis of movement (Fig 7) revealed a stark contrast between crabs isolated for 3 hours and all other groups. While crabs in the control and short-term isolation groups were active throughout the trial, the 3-hour isolation group remained almost completely immobile during the initial phase. This was followed by a phase of hyperactivity, where their movement distance dramatically increased.

**Fig 8:**
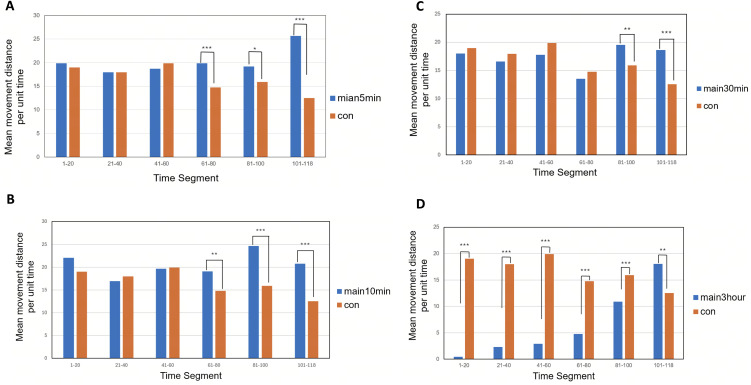
Histogram of the moving distance of individuals under different isolation durations compared to the control experiment. (A) 5-minute isolation. (B) 10-minute isolation. (C) 30-minute isolation. (D) 3-hour isolation.

The 3-hour isolation group showed a unique biphasic response. During the initial phase (1–20), they remained almost completely immobile, with movement distances significantly lower than the control group (t(19) = −17.44, p < 0.001, Cohen’s d = −5.63), corresponding to an approximate 97.6% decrease compared to controls (Fig 8D). This was followed by a shift to hyperactivity, where their movement in the final time segment (101–118) significantly exceeded that of controls (t(17) = 3.22, p = 0.005, Cohen’s d = 1.13), corresponding to an approximate 43.9% increase (Fig 8D).

**Fig 9:**
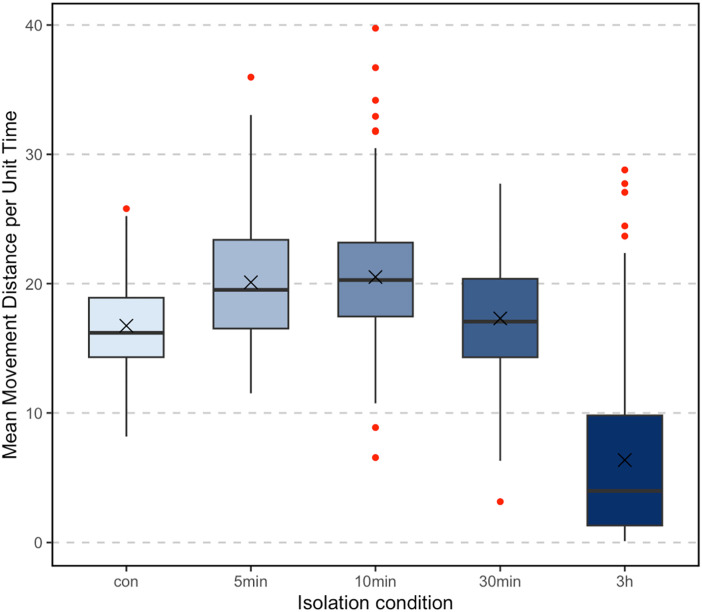
Box plots of moving distance under 5-, 10-, 30-minute, and 3-hour isolation conditions and the control experiment.

In contrast, crabs isolated for shorter durations (5, 10, and 30 minutes) generally displayed greater movement than the control group throughout the trial (Fig 9). For instance, the 5-minute isolation group exhibited significantly higher movement starting from the 61–80 interval (t(19) = 4.72, p < 0.001, Cohen’s d = 1.35), corresponding to an approximate 34.6% increase, and continuing through the end of the trial (101–118: t(17) = 10.74, p < 0.001, Cohen’s d = 3.49), corresponding to an approximate 104.8% increase (Fig 8A). Similar patterns were observed for the 10-minute group (101–118: t(17) = 8.37, p < 0.001, Cohen’s d = 2.97), corresponding to an approximate 65.6% increase (Fig 8B) and the 30-minute group (101–118: t(17) = 7.65, p < 0.001, Cohen’s d = 2.42), corresponding to an approximate 48.8% increase (Fig 8C).

#### Distance to nearest neighbor.

**Fig 10:**
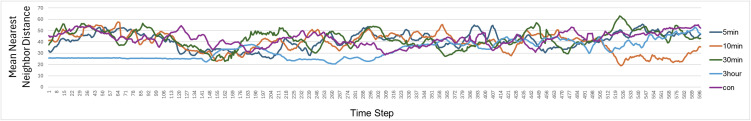
Time course of nearest-neighbor distance under different isolation durations and in the control condition. Curves are color-coded by isolation duration (5, 10, 30 minutes, 3 hours) and by the control condition.

**Fig 11:**
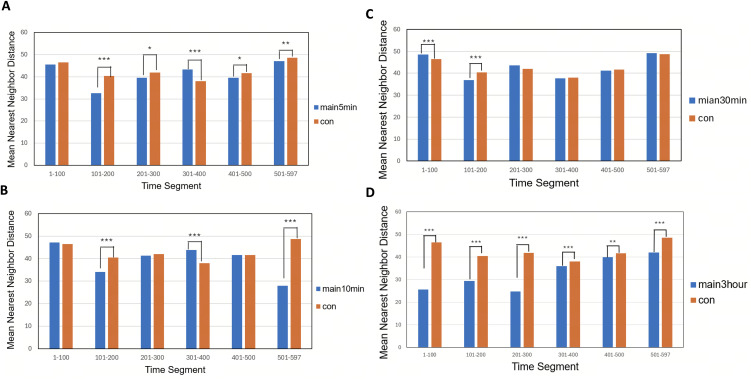
Histogram of the distance to the nearest neighbor under different isolation durations compared to the control experiment. (A) 5-minute isolation. (B) 10-minute isolation. (C) 30-minute isolation. (D) 3-hour isolation.

Social proximity was strongly influenced by isolation duration (Fig 10). The 3-hour isolation group maintained a significantly smaller distance to neighbors during the initial immobility phase (1–100: t(99) = −49.14, p < 0.001, Cohen’s d = −6.76), corresponding to an approximate 44.9% reduction relative to controls (Fig 11D). As they entered the hyperactive phase, this distance remained lower than controls until the final segment (501–597: t(96) = −14.13, p < 0.001, Cohen’s d = −1.38), corresponding to an approximate 13.4% reduction (Fig 11D).

For shorter durations, the response was non-monotonic. The 10-minute isolation group showed clear compensatory social seeking, with significantly closer proximity to neighbors in the final segment (501–597: t(96) = −27.20, p < 0.001, Cohen’s d = −4.43), corresponding to an approximate 42.6% reduction (Fig 11B). However, the 5-minute group (301–400: t(99) = 6.98, p < 0.001, Cohen’s d = 1.04), corresponding to an approximate 14.0% increase, and the 30-minute group (1–100: t(99) = 3.94, p < 0.001, Cohen’s d = 0.42), corresponding to an approximate 4.3% increase (Fig 11A, 11C), exhibited significantly larger nearest-neighbor distances in several time segments.

**Fig 12:**
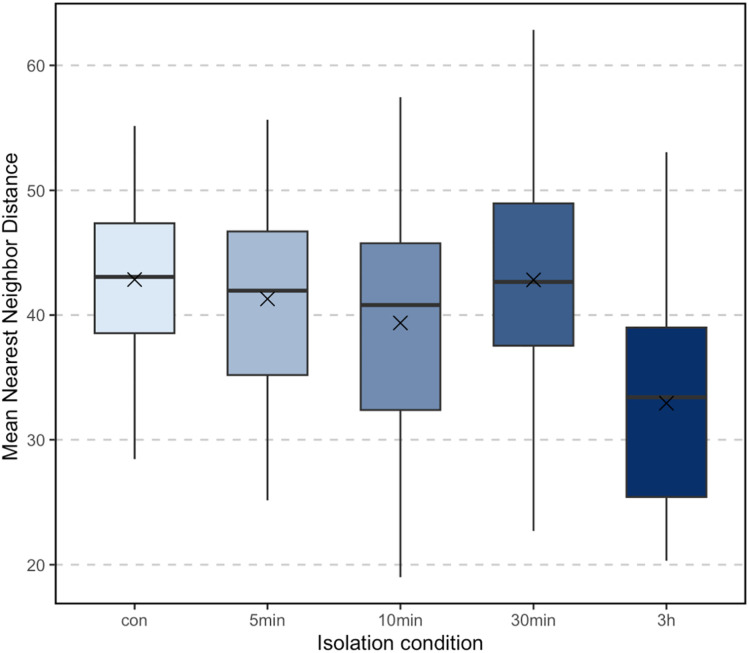
Box plots of the distance to the nearest neighbor under 5-, 10-, 30-minute, and 3-hour isolation conditions and the control experiment.

These patterns are further supported by the distributional comparisons shown in Fig 12.

#### Local polarity.

**Fig 13:**
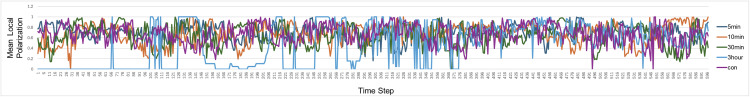
Mean local polarization over time under different isolation durations and in the control condition. The radius was set to 200 normalized distances, and curves are color-coded by isolation duration and by the control condition.

**Fig 14:**
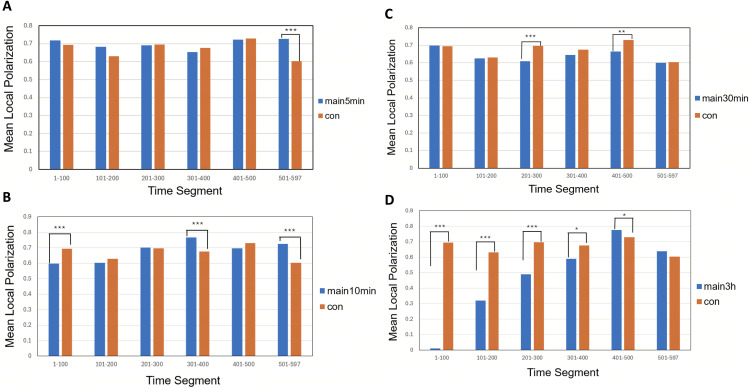
Histogram of local polarization under different isolation durations compared to the control experiment. (A) 5-minute isolation. (B) 10-minute isolation. (C) 30-minute isolation. (D) 3-hour isolation.

Similar to movement distance and social proximity, the time course of local polarity revealed duration-dependent differences in movement coordination (Fig 13). Local polarity in the 3-hour isolation group was near zero during the initial phase (1–100: t(99) = −42.19, p < 0.001, Cohen’s d = −5.59), corresponding to an approximate 98.6% reduction relative to controls (Fig 14D), and remained significantly lower than controls through the 301–400 interval (t(99) = −2.21, p = 0.030, Cohen’s d = −0.29), corresponding to an approximate 12.8% reduction (Fig 14D). In the 401–500 interval, polarity was significantly higher than controls (t(99) = 2.18, p = 0.032, Cohen’s d = 0.26), corresponding to an approximate 6.2% increase (Fig 14D), suggesting a late recovery in movement coordination.

Among the short-term isolation groups, the 10-minute group showed significantly lower polarity in the initial segment (1–100: t(99) = −4.01, p < 0.001, Cohen’s d = −0.53), corresponding to an approximate 14.0% reduction, followed by significantly higher polarity in later segments (301–400: t(99) = 4.04, p < 0.001, Cohen’s d = 0.55; 501–597: t(96) = 3.79, p < 0.001, Cohen’s d = 0.56), corresponding to approximately 13.6–20.3% increases (Fig 14B). The 5-minute group showed significantly higher polarity only in the final segment (501–597: t(96) = 5.06, p < 0.001, Cohen’s d = 0.72), corresponding to an approximate 20.5% increase (Fig 14A). The 30-minute group showed significantly lower polarity in the 201–300 and 401–500 intervals (t(99) = −3.42, p < 0.001, Cohen’s d = −0.49; t(99) = −2.84, p = 0.006, Cohen’s d = −0.36), corresponding to approximately 12.4–8.9% reductions (Fig 14C), with no other significant differences (see S1 Table for full statistics).

## Discussion

Our results provide clear experimental evidence that enforced social isolation leads to significant, duration-dependent alterations in collective behavior after reintegration into the group. These effects appear to arise from isolation-induced modulation of individual behavior that propagates through local interactions. The primary finding is that while short-term isolation (<30 min) triggers compensatory social-seeking behaviors (i.e., hyperactivity and increased proximity), long-term isolation (3 h) induces a severe biphasic response: an initial period of immobility and social withdrawal, followed by a phase of poorly coordinated hyperactivity. This non-linear effect suggests the existence of a critical threshold beyond which the costs of social deprivation escalate, leading to pronounced disruptions in the reintegration process rather than immediate or efficient reintegration into the group.

The increased locomotor activity and reduced inter-individual distance observed after short-term isolation are consistent with compensatory social-seeking behavior reported across taxa [9,11]. For example, transient isolation in rodents has been shown to enhance social approach and exploratory behavior [9], while in social insects, temporary separation can induce increased interaction rates upon reunion [11]. These findings suggest that short-term isolation induces a reversible shift in internal state that promotes rapid reintegration into social groups. Importantly, the effects of short-term isolation were not monotonic across durations. While individuals isolated for 10 minutes showed a clear reduction in nearest-neighbor distance, consistent with compensatory social-seeking behavior, crabs isolated for slightly shorter (5 min) or longer (30 min) durations exhibited increased inter-individual distances during parts of the trial. This non-linear pattern suggests that compensatory responses may be restricted to a narrow temporal window, whereas isolation durations outside this window may transiently disrupt local coordination without inducing the pronounced dysfunction observed after prolonged isolation.

In contrast, prolonged isolation produced a qualitatively different response characterized by an initial phase of behavioral suppression followed by disorganized hyperactivity. The behavioral phase transition observed after 3 hours of isolation can be interpreted as a breakdown in the ability to process and respond to social cues. The initial immobility may reflect a transient dysfunctional state that limits immediate social engagement, while the subsequent hyperactivity—which remained poorly coordinated—suggests impaired adjustment to neighboring individuals. Notably, polarity in the 3-hour group recovered to above-control levels in the 401–500 interval, suggesting that coordination was eventually re-established, albeit with a substantial delay. This confirms that isolation alters individual motivational states and can locally perturb interaction dynamics, resulting in detectable changes in collective behavior without implying control over the entire swarm.

The identification of a behavioral threshold between short and long isolation durations is particularly noteworthy. Rather than a linear accumulation of effects, our results indicate a transition point beyond which the system shifts into a qualitatively different dynamical regime. Similar threshold-like responses have been reported in collective systems, where small changes in individual parameters can lead to abrupt transitions in group-level organization [18,19]. This suggests that isolation may modulate effective interaction rules, thereby altering the stability of collective states. From a theoretical perspective, these findings are consistent with frameworks in which collective behavior emerges from the interplay between individual internal states and local interaction rules [3,16]. In this view, isolation does not merely affect individual activity levels but reshapes the effective interaction landscape, thereby influencing emergent group dynamics.

Similar non-linear or threshold-like responses to social isolation have been reported across taxa, including insects and vertebrates [5,7], where short-term deprivation induces compensatory social behaviors while longer isolation leads to withdrawal, dysregulation, or impaired coordination. Similar biphasic or non-linear responses to social deprivation have been reported in vertebrates, where extended isolation leads to dysregulation of stress systems and impaired social processing [5,7]. Our study extends these findings to an anonymous collective system, demonstrating a clear dose-dependent effect in an anonymous swarm where the tendency to re-establish collective organization is quantitatively reflected in movement patterns. This suggests that threshold-dependent responses to social disruption may represent a general feature of collective and social systems [2,38], rather than being limited to cognitively complex organisms, but instead emerging from more general interaction-based mechanisms.

These findings carry significant ecological implications. Impaired coordination following prolonged isolation could increase predation risk and reduce foraging efficiency for the entire group. While prolonged isolation and deliberate reintegration may be relatively rare in natural settings, transient separation of individuals from swarms can occur due to environmental heterogeneity, tidal dynamics, or physical barriers on intertidal flats. In this context, our experimental design captures a simplified but ecologically relevant scenario in which temporarily isolated individuals subsequently encounter and rejoin conspecific groups. Rather than replicating natural conditions in full, the study aims to isolate the behavioral consequences of transient social disruption under controlled conditions, and the distinct biphasic response observed here highlights how even brief social disruption can shape reintegration dynamics in ways that are ecologically consequential.

An important limitation of the present study is that isolation durations between 30 minutes and 3 hours were not sampled. Future studies should therefore examine intermediate time scales within this interval to determine whether the observed transition reflects a gradual shift or a sharper behavioral threshold. While our study provides robust insights under controlled laboratory conditions, we acknowledge that these settings do not fully replicate the complex environmental pressures of the crabs’ natural habitat; future research could validate these findings in semi-natural settings to incorporate factors such as tidal cycles and predation threats. Future work could also investigate the proximate mechanisms mediating these behavioral shifts, such as the role of stress hormones (e.g., crustacean hyperglycemic hormone). Together, these directions would further establish *Mictyris guinotae* as a tractable invertebrate model for exploring the fundamental behavioral dynamics of social reintegration and the mechanisms by which individual-level perturbations propagate through local interactions to shape emergent collective organization.

## Conclusions

This study demonstrates that social isolation has significant, duration-dependent impacts on the reintegration behavior of *Mictyris guinotae* within a swarm. We identify a critical threshold between 30 minutes and 3 hours of isolation, beyond which behavioral responses shift from compensatory increases in activity and social proximity to a biphasic pattern of withdrawal followed by poorly coordinated hyperactivity.

These findings quantify the resilience of an anonymous collective to transient social perturbation and show how isolation-induced changes in individual behavior are reflected in emergent group-level dynamics. Together, our results establish *M. guinotae* as a valuable model system for investigating how disruptions at the individual level propagate through local interactions to influence collective organization.

## Supporting information

S1 Table Statistical results for Experiment 1 and Experiment 2. Legend for S1 Table (Supplementary Table 1). This file contains the detailed statistical results for Experiment 1 and Experiment 2 as reported in the manuscript. The tables provide the outputs of t-tests comparing behavioral parameters between the experimental (isolated) and control groups across different time segments. These results correspond to the significance markers shown in figures of the main text. File naming convention: s1_experiment1: Statistical results for Experiment 1 (corresponds to Figs 4 and 6). s1_experiment2: Statistical results for Experiment 2 (corresponds to Figs 8, 11 and 14). Column definitions: Experiment: Experimental condition analyzed (e.g., “Exp 1”, “Exp 2 (5 min)”). Parameter (Figure): Behavioral metric tested and corresponding figure in the main text. Time Segment: Time interval used for each test (e.g., “1–50”). Statistical Test: Statistical method employed (t-test). Mean (Main): Mean of the experimental (isolated) group. Variance (Main): Variance of the experimental group. SD (Main): Standard deviation of the experimental group. Mean (Control): Mean of the control group. Variance (Control): Variance of the control group. SD (Control): Standard deviation of the control group. t-value: Test statistic. df: Degrees of freedom. p-value: Resulting p-value.
(XLSX)

S1 Data Coordinate data for Experiment 1 and Experiment 2. Data Legend for Supplementary Data File. File Contents: This supplementary file contains the raw coordinate data from the tracking experiments described in the manuscript. The data is provided in wide format, with each sheet corresponding to a single experimental trial. Sheet/File Naming Convention: experiment1_main: Experiment 1, isolated individual trial (12 crabs total). experiment1_con: Experiment 1, control individual trial (12 crabs total). experiment2_5 min: Experiment 2, 5-minute group isolation trial (20 crabs total). experiment2_10 min: Experiment 2, 10-minute group isolation trial (20 crabs total). experiment2_30 min: Experiment 2, 30-minute group isolation trial (20 crabs total). experiment2_3h: Experiment 2, 3-hour group isolation trial (20 crabs total). experiment2_con: Experiment 2, control group trial (20 crabs total). Data Structure (within each sheet): Rows: Each row represents a single time step. Columns: Coordinates are grouped by individual crab, labeled “NO.1” through “NO.12” (for Exp 1) or “NO.20” (for Exp 2). NO.1_X: X-coordinate (in pixels) for Crab 1. NO.1_Y: Y-coordinate (in pixels) for Crab 1. NO.2_X: X-coordinate (in pixels) for Crab 2. NO.2_Y: Y-coordinate (in pixels) for Crab 2 and so on for all crabs in the trial.
(XLSX)

S1 Methods Formal definitions of behavioral metrics. This supplementary file contains the formal mathematical definitions of the behavioral metrics used for quantitative analysis in the main text. These definitions support analytical transparency and reproducibility and complement the descriptive explanations provided in the Materials and Methods.
(DOCX)
