# Supplementary material for: Duration-dependent effects of social isolation on reintegration behavior in the swarming soldier crab, Mictyris guinotae
Source: PLoS One. 2026 Jul 1;21(7):e0350642. doi: 10.1371/journal.pone.0350642 (PMC13322545; doi:10.1371/journal.pone.0350642)
Supplement: S1 Methods — This supplementary file contains the formal mathematical definitions of the behavioral metrics used for quantitative analysis in the main text. These definitions support analytical transparency and reproducibility and complement the descriptive explanations provided in the Materials and Methods. (DOCX) [file pone.0350642.s003.docx]

This section provides the formal mathematical definitions of the behavioral metrics used in the main text. These definitions are provided to ensure analytical transparency and reproducibility and supplement the descriptive explanations given in the Materials and Methods.

**Distance to the nearest neighbor**

The first metric, $D_{k}^{t},$ is the distance to the nearest neighbor of the $k$-th individual at each time step, $t$, and that is defined by

$D_{k}^{t}=\min\{\sqrt{{(x_{k}^{t}-x_{s}^{t})}^{2}+{(y_{k}^{t}-y_{s}^{t})}^{2}}, s\in\{1, 2, \ldots, N\}\}$,

where $\min S$ with a set $S$ represents the least element, $\{1, 2, \ldots, N\}$ represents the individual number, and $(x_{k}^{t}, y_{k}^{t})$ represents the co-ordinate of $k$-th individual at time step, $t$.

**Local polarity**

The second metric, $P_{k}^{t}$, is the local polarization which represents the degree of velocity matching at a local site, where the velocity is defined per unit time, $\tau$, and that is defined for the $k$-th individual at each time step by

$$P_{k}^{t}=\sqrt{\left( \frac{\sum_{p} u_{p}^{t}}{{n(r)}_{k}^{t}} \right)^{2}+\left( \frac{\sum_{p} w_{p}^{t}}{{n(r)}_{k}^{t}} \right)^{2}}$$

where $u_{p}^{t}$ and $w_{p}^{t}$ are defined for each swarm-mate, $p$ at time step, $t$, involved in the $k$-th individual’s neighborhood, and $n_{k}^{t}(r)$ represents the number of swarm-mates involved in the $k$-th individual’s neighborhood with radius $R$. Thus, $u_{p}^{t}$ and $w_{p}^{t}$ are defined by

$$u_{p}^{t}=\frac{(x_{p}^{t}-x_{p}^{t-\tau})}{\sqrt{{(x_{p}^{t}-x_{p}^{t-\tau})}^{2}+{(y_{p}^{t}-y_{p}^{t-\tau})}^{2}}}$$

$$w_{p}^{t}=\frac{(y_{p}^{t}-y_{p}^{t-\tau})}{\sqrt{{(x_{p}^{t}-x_{p}^{t-\tau})}^{2}+{(y_{p}^{t}-y_{p}^{t-\tau})}^{2}}}$$

if ${(x_{p}^{t}-x_{p}^{t-\tau})}^{2}+{(y_{p}^{t}-y_{p}^{t-\tau})}^{2}>0$, and $u_{p}^{t}=w_{p}^{t}=0,$ otherwise, where the swarm-mate, $p$ at time step, $t$ satisfies the condition such that

$\sqrt{{(x_{p}^{t}-x_{k}^{t})}^{2}+{(y_{p}^{t}-y_{k}^{t})}^{2}}<R$.

**Movement distance over time**

The third metric, $M_{k}^{t}$, is the moving distance of the $k$-th individual at each time step, $t$, within a $F$ times unit time, and that is defined by

$M_{k}^{t}=\sum_{i=0}^{F-1} \sqrt{{(x_{k}^{t-i\tau}-x_{k}^{t-(i+1)\tau})}^{2}+{(y_{k}^{t-i}-y_{k}^{t-(i+1)\tau})}^{2}}$.

By definition, if all individuals within the neighborhood remain immobile, then for all $p$, $u_{p}^{t}=w_{p}^{t}=0,$ yielding $P_{k}^{t}=0.$ If only a single individual within the neighborhood moves while all others remain immobile, then only that individual contributes to the summation, and $P_{k}^{t}$ takes a value of 1. In this limiting case,

$P_{k}^{t}=\sqrt{\frac{{(x_{s}^{t}-x_{s}^{t-\tau})}^{2}}{{(x_{s}^{t}-x_{s}^{t-\tau})}^{2}+{(y_{s}^{t}-y_{s}^{t-\tau})}^{2}}+\frac{{(y_{s}^{t}-y_{s}^{t-\tau})}^{2}}{{(x_{s}^{t}-x_{s}^{t-\tau})}^{2}+{(y_{s}^{t}-y_{s}^{t-\tau})}^{2}}}=1$.
